# The redox sensitive glycogen synthase kinase 3β suppresses the self-protective antioxidant response in podocytes upon oxidative glomerular injury

**DOI:** 10.18632/oncotarget.6303

**Published:** 2015-11-11

**Authors:** Changbin Li, Yan Ge, Ai Peng, Rujun Gong

**Affiliations:** ^1^ Department of Nephrology, Shanghai Tenth People's Hospital, Tongji University School of Medicine, Shanghai, China; ^2^ Division of Kidney Disease and Hypertension, Department of Medicine, Rhode Island Hospital, Brown University School of Medicine, Providence, Rhode Island, USA

**Keywords:** conditional gene knockout, glycogen synthase kinase 3β, podocyte, glycogen, antioxidant defense, Pathology Section

## Abstract

The redox sensitive glycogen synthase kinase (GSK) 3 has been recently implicated in the pathogenesis of proteinuric glomerulopathy. However, prior studies are less conclusive because they relied solely on chemical inhibitors of GSK3, which provide poor discrimination between the isoforms of GSK3 apart from potential off target activities. In murine kidneys, the β rather than the α isoform of GSK3 was predominantly expressed in glomeruli and distributed intensely in podocytes. By employing the doxycycline-activated Cre-*lox*P site specific gene targeting system, GSK3β was successfully knocked out (KO) selectively in podocytes in adult mice, resulting in a phenotype no different from control littermates. Electron microscopy of glomeruli in KO mice demonstrated more glycogen accumulation in podocytes but otherwise normal ultrastructures. Upon oxidative glomerular injury induced by protein overload, KO mice excreted significantly less albuminuria and had much attenuated podocytopathy and glomerular damage. The anti-proteinuric and glomerular protective effect was concomitant with diminished accumulation of reactive oxygen species in glomeruli in KO mice, which was likely secondary to a reinforced Nrf2 antioxidant response in podocytes. Collectively, our data suggest that GSK3β is dispensable for glomerular function and histology under normal circumstances but may serve as a therapeutic target for protecting from oxidative glomerular injuries.

## INTRODUCTION

Glycogen synthase kinase (GSK) 3 is a highly-conserved, ubiquitously expressed, and constitutively active serine/threonine protein kinase that was originally identified in 1980 as a key cellular signaling transducer involved in glycogenesis and mediating inhibitory phosphorylation of glycogen synthase [1]. GSK3 takes center stage more than 20 years after its discovery, when interest in GSK3 expanded greatly well beyond glycogen metabolism to tumorigenesis, cell-cycle progression, cytoskeletal organization, development control, inflammation and immunity, mitochondria permeability transition, adaptive response to oxidative stress, and more [2–4]. In mammals, GSK3 exists as two isoforms, GSK3α (51 kDa) and GSK3β (47 kDa), which are encoded by separate genes that produce highly homologous proteins with significant difference only in their N- and C-terminal regions [5]. Although displaying 84% structural homology, GSK3α and GSK3β are not functionally interchangeable, and GSK3β possesses unique biological actions. In support of this, knockout of the GSK3β gene in mice proves to be lethal in the embryonic stage with no rescue by the intact α isoform of GSK3, leading to the notion that the β isoform may play an important role in cell growth and differentiation [6]. In accordance, GSK3β instead of GSK3α has been shown to regulate cardiac development and cardiomyocyte proliferation [7]. GSK3 is situated at the nexus of numerous signaling pathways, and dictates the activity of many signaling transducers and transcription factors as its cognate substrate, such as RelA/p65, β-catenin and cyclin D1 [1, 8–10]. Moreover, GSK3 is also a redox sensitive signaling molecule and plays a pivotal role in controlling the self-protective antioxidant defense. This effect is achieved through promoting the nuclear export and degradation of nuclear factor erythroid 2-related factor 2 (Nrf2) upon oxidative stress, culminating in the switching off the Nrf2 antioxidant response [11–14].

Recent evidence suggests that GSK3β plays an important role in the pathogenesis of glomerular podocytopathy and proteinuria [15–18]. However, most of the previous findings relied on the use of small molecule inhibitors of GSK3β, which have poor selectivity between the α and β isoforms of GSK3; furthermore, use of chemical inhibitors *in vivo* is unable to discriminate a direct primary podocyte effect from that secondary to a systemic effect. Thus, previous studies are not adequate to conclusively evaluate the exact role of GSK3β in podocyte physiology and pathology. Genetic disruption of GSK3β would be more selective but unfortunately cause embryonic lethality [6]. To circumvent this limitation, it is imperative to establish a mouse model in which the podocyte-specific expression of GSK3β is selectively deleted. In view of the indispensable role of GSK3β related cell signaling in embryonic development, inducible deletion of GSK3β in mature glomerular podocytes will be an ideal approach to avoid potential congenital defects of the kidney.

The Cre/*lox*P site specific recombination system is a tool for tissue-specific and with the *tet* system, time-specific, gene targeting that cannot be investigated by conventional knockout due to embryonic lethality. In this study, we employed the doxycycline inducible Cre/*lox*P gene targeting system to specifically delete GSK3β in podocytes in adult mice. To restrict deletion of floxed GSK3β to mature podocytes in adult mice, we have utilized a conditional inducible expression system (Tet-On) in which GSK3β can be deleted in a time- and cell-specific manner. In this system, the reverse tetracycline-controlled transcriptional activator (*rtTA*) is expressed under the control of the podocyte-specific podocin promoter (*NPHS2*), such that rtTA is only produced in kidney podocytes. A second transgene uses the *tet*O promoter elements upstream of a minimal CMV promoter to drive expression of Cre recombinase. The effects of podocyte-specific knockout of GSK3β were examined in mice under physiologic condition and in a murine model of protein overload induced podocyte injury and proteinuria.

## RESULTS

### The β isoform of GSK3 is predominantly expressed in glomerular podocytes *in vivo* and *in vitro*

**Figure 1:**
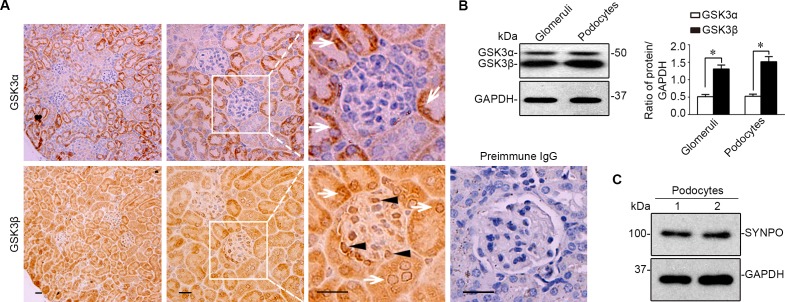
The β instead of the α isoform of GSK3 is predominantly expressed in glomeruli and mainly located to podocytes in mouse kidneys **A.** Representative micrographs of peroxidase immunohistochemistry staining of mouse kidney specimens for GSK3α and GSK3β. Arrows indicate GSK3α- or GSK3β-positive tubular cells. Arrowheads show the staining of GSK3α or GSK3β in glomerular cells. As a negative control, the primary antibody was replaced by preimmune IgG and no specific staining was noted. Scale bar = 20μm. **B.** Homogenates of glomeruli isolated from mice and lysates of primary podocytes were subjected to immunoblot analysis for GSK3α/β and GAPDH. Relative abundance of GSK3 and GSK3β expressed in murine glomeruli and primary podocytes determined by densitometric analyses of immunoblot. *P <0.01, (*n* = 4, unpaired *t*-tes­­­­t). **C.** Immunoblot analysis demonstrates that primarily cultured podocytes evidently express synaptopodin, a typical marker of podocytes.

GSK3 has been described as a ubiquitously expressed kinase involved in multiple cellular signaling pathways [1, 19, 20]. Nevertheless, its expression in the kidney has been understudied. Recent evidence suggests that the α and β isoforms of GSK3 are not equally expressed in certain fragments of distal nephrons [21]. However, the expression pattern of the two isoforms in glomerulus remains unknown. To this end, consecutive sections of formalin fixed paraffin embedded murine kidney specimens were processed for peroxidase immunohistochemistry staining for GSK3α or GSK3β in parallel. Shown in Figure 1A, both the α and the β isoforms of GSK3 were noted to be expressed in parenchymal kidney tissues. In glomeruli, the β rather than the α isoform was intensely detected. High power light microscopy indicated that the staining of GSK3β was mainly distributed to the periphery of glomerular tufts, consistent with podocyte localization. To corroborate the morphologic findings, glomeruli were isolated from mouse kidneys by the magnetic beads based approach and homogenized (Figure 1B) or processed for primary culture of podocytes, which were characterized and verified to express typical podocyte marker proteins, like synaptopodin (Figure 1C). Immunoblot analysis of homogenates of isolated glomeruli or primary podocyte lysates demonstrated much more abundant expression of GSK3β than GSK3α (Figure 1B), as probed by a monoclonal antibody that recognizes both GSK3α and GSK3β.

### The doxycycline inducible deletion of GSK3β in mature glomerular podocytes in adult mice results in a healthy phenotype with normal kidney function

**Figure 2:**
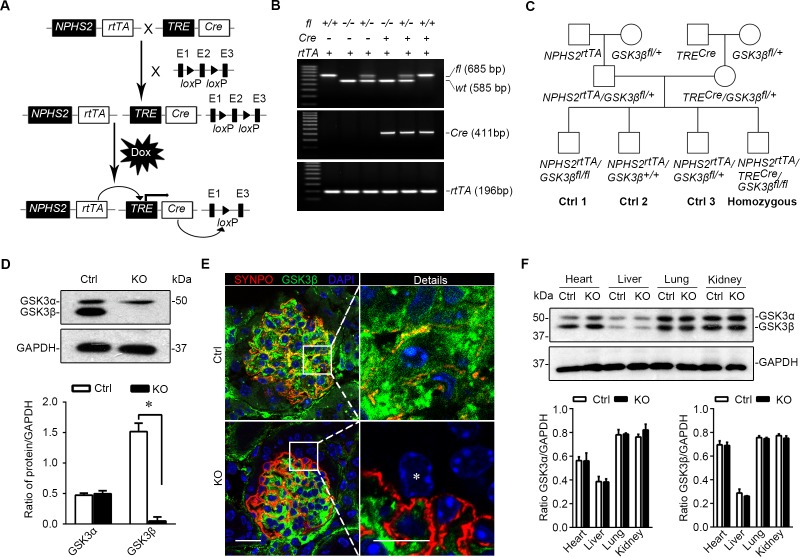
Mice with podocyte specific deletion of GSK3β are generated by the doxycycline inducible Cre/*lox*P mediated gene targeting system **A.** Schematic diagram depicts the breeding strategy to generate the inducible podocyte-specific *GSK3β* gene knockout mice. The deletion of exon 2 (E2) in the event of recombination of the *GSK3β* gene is achieved with the addition of doxycycline (Dox). **B.** Representative images showing PCR analysis of the genomic DNA extracted from the clipped tail tissues. The PCR bands of wild-type (*wt*, 585 bp), floxed (*fl*, 685bp) *GSK3β*, *Cre* (411bp) and *rtTA* (196bp) are indicated. **C.** Breeding pedigrees to generate control (*Cre*-negative) and homozygous (triple transgenic) mice. **D.** Immunoblot analysis for GSK3α and GSK3β in primary podocytes derived from KO mice or Ctrl littermates (16 weeks old). Note that Ctrl podocytes express both GSK3α and GSK3β, whereas expression of GSK3β was successfully ablated in KO podocytes. Relative abundance of GSK3α and GSK3β expressed in primary podocytes determined by densitometric analyses of immunoblot. *P <0.01 (*n* = 3, unpaired *t*-tes­­­­t). **E.** Dual color fluorescence immunohistochemistry staining of kidney sections from Ctrl or KO mice for SYNPO (red) and GSK3β (green). Sections were counterstained with DAPI (blue). *indicates the nucleus of a podocyte in the glomerulus of KO mice, where GSK3β has been completely ablated. Left panel, scale bar = 20μm. Right panel (Details), scale bar = 10μm. **F.** Protein expression of GSK3α and GSK3β was not affected in the heart, liver, lung, and whole kidney tissues in KO mice as compared with Ctrl mice (16 weeks old). Relative abundance of GSK3α and GSK3β expressed in heart, liver, lung and kidney from Ctrl (white bar) and KO (black bar) mice determined by densitometric analyses of immunoblot. Not statistically significant between Ctrl and KO mice (*n* = 3, unpaired *t*-test).

To understand the role of GSK3 in podocyte pathobiology, we next chose to knockout GSK3β, the major isoform of GSK3 expressed in glomerular podocytes. In line with the pivotal role of GSK3β in embryo development, systemic GSK3β knockout would cause embryonic lethality [6] and thus seems not to suit our purpose. The doxycycline-inducible Cre/*lox*P mediated gene targeting (Figure 2A) permits both spatial and temporal control of the target gene expression and thus was employed to target GSK3β specifically in podocytes in adult mice. By crossing the *GSK3β*-floxed mice (*GSK3β^fl/fl^*) with transgenic mice expressing *rtTA* under the control of podocin promoter (*NPHS2*) and those expressing *Cre* driven by the *tet*O-CMV promoter (Figure 2A), progeny with the genotype of doxycycline-inducible podocyte-specific GSK3β knockout on a FVB genetic background were successfully bred and confirmed by genotyping tail tissues (Figure 2B) to carry both *Cre* and *rtTA* transgenes and homozygous floxed-GSK3β (*NPHS2^rtTA^/TRE^Cre^/GSK3β^fl/fl^*), which were designated as knockout (KO) mice in subsequent studies. All littermates lacking the *Cre* transgene were designated as control mice (Figure 2C). After oral administration of doxycycline for 2 weeks, the expression of GSK3β in glomerular podocytes was substantially reduced in KO mice as proved by immunoblot analysis of primarily cultured podocytes for GSK3β (Figure 2D). Dual color fluorescent immunohistochemistry staining of frozen kidney specimens for synaptopodin and GSK3β demonstrated that the staining of GSK3β was evidently diminished in synaptopodin positive podocytes that were located mainly in the periphery of glomerular tufts (Figure 2E), indicative of a successful podocyte-specific knockout. Because glomeruli account for only ~2% of the total kidney mass [22], podocyte selective ablation of GSK3β barely affected the abundance of GSK3α or GSK3β in total kidney homogenates as estimated by immunoblot analysis (Figure 2F). Likewise, the expression profile of GSK3 isoforms in other organ systems, including heart, liver and lung, was not altered in KO mice (Figure 2F), confirming a glomerular podocyte specific ablation.

**Figure 3:**
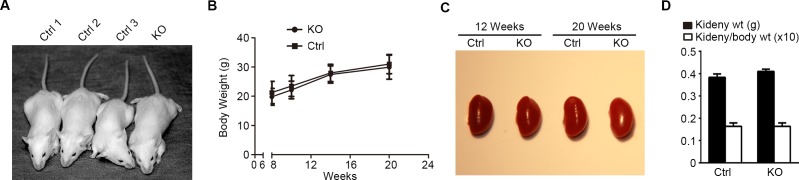
Loss of GSK3β in podocytes results in no noticeable difference in gross appearance of mice **A.** Representative images of control (Ctrl) and podocyte-specific GSK3β knockout (KO) mice show no difference in gross appearance, development, and behavior at twenty weeks of age. **B.** Body weight curve for a representative litter of sex-matched (male) knockout mice (solid circle) and control littermates (solid square); not statistically significant at all observed time points (*n* = 6). **C.** A representative litter of sex-matched (male) KO and Ctrl mice had normal and comparable kidneys in terms of gross appearance, size, and color. **D.** KO mice and Ctrl littermates had similar and normal kidney weights and kidney to body weight ratios; not statistically significant between the two groups (*n* = 6).

**Figure 4:**
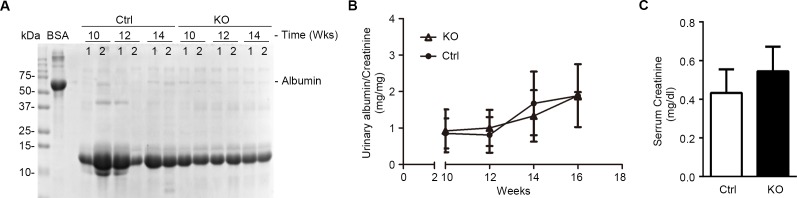
Mice with podocyte-specific deletion of GSK3β have normal kidney physiology **A.** Spot urine was collected at the indicated time points and was subjected to SDS-PAGE followed by Coomassie Brilliant Blue staining. BSA (10μg) served as a standard control. Urine samples (5μl) collected on the indicated time points from each group were loaded. **B.** Quantification of urine albumin levels adjusted with urine creatinine concentrations; not statistically significant between the two groups, KO (open triangle) *versus* Ctrl littermates (solid triangle) (*n* = 6). **C.** Blood sample from 16-week old KO and Ctrl mice was subjected to serum creatinine assay; not statistically significant between the two groups (*n* = 6).

Except the deletion of GSK3β in glomerular podocytes at a molecular level, KO mice were otherwise not different from control littermates in terms of behavior and development (Figure 3A), as reflected by growth curves for body weight (Figure 3B). Moreover, KO and control mice also presented comparable gross kidney morphology (Figure 3C) and kidney to body weight ratios (Figure 3D). Podocytes are a crucial structural component of the glomerular filtration barrier. To determine the impact of podocyte loss of GSK3β on glomerular filtration barrier function, albuminuria was examined by urine electrophoresis (Figure 4A) and quantified by urinary albumin to creatinine ratios (Figure 4B). Under normal conditions, KO mice and control littermates excreted equal and scarce amounts of low-molecular-weight proteins in urine, which are consistent with the small-sized proteins normally secreted by tubular epithelia; no albumin or higher-molecular-weight proteins were observed in both control and KO mice at indicated ages under normal conditions, suggesting that KO mice are less likely to have glomerular sieving defect. Furthermore, the serum creatinine levels were also noted to be same in KO and control mice (Figure 4C), denoting a normal kidney function following GSK3β knockout in podocytes.

### Mice with podocyte specific ablation of GSK3β present normal kidney histology except an increase of glycogen accumulation in podocytes

**Figure 5:**
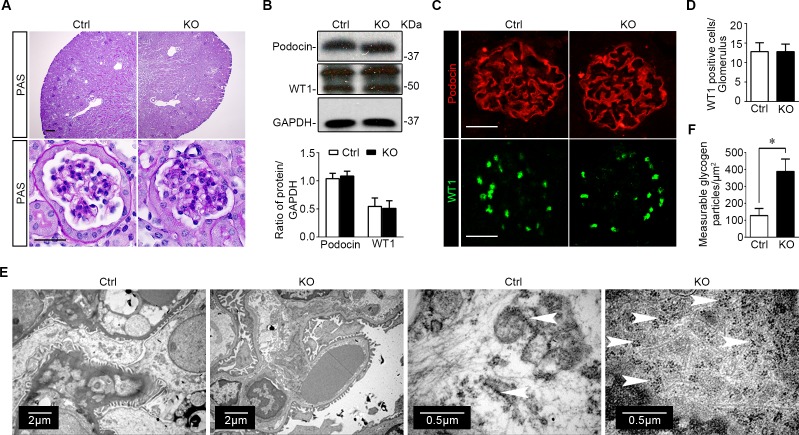
Specific ablation of GSK3β in mature glomerular podocytes does not affect kidney histology except an increase of glycogen accumulation in podocytes **A.** Representative micrographs of PAS staining of kidney specimens procured from KO mice and Ctrl littermates at 16 weeks old; Scale bar = 20μm. **B.** Immunoblot analysis of glomeruli isolated from KO and Ctrl mice showed equal expression of podocin, WT1, and GAPDH. Relative abundance of WT1 and podocin expressed in isolated glomeruli from Ctrl (white bar) and KO (black bar) mice determined by densitometric analyses of immunoblot. Not statistically significant between Ctrl and KO mice (*n* = 3, unpaired *t*-test). **C.** Representative micrographs of fluorescent immunohistochemistry staining of kidney cryosections for podocyte slit diaphragm protein podocin and the podocyte-specific marker WT1; Scale bar = 20μm. **D.** Absolute counting of WT1 positive podocytes in each glomerulus demonstrated that the number of podocytes per glomerulus was comparable between control and KO mice. Not statistically significant between the two groups (*n* = 6, unpaired *t*-test). **E.** Representative transmission electron microscopic images of glomeruli showed that the ultrastructures of glomerulus and podocytes were similar in KO and Ctrl mice, whereas increased deposition of glycogen particles was found in glomerular podocytes in KO mice. White arrowheads indicate glycogen particles, which appear as electron-dense particles ranging in size from 20 to 25nm in diameter and closely associated with the network of tubules of the smooth endoplasmic reticulum. **F.** Computerized morphometric quantification of glycogen particles showed that the amount of measurable glycogen particles was augmented in glomerular podocytes in KO mice.*P <0.01 (*n* = 6, unpaired *t*-test).

Kidney specimens procured from KO and control mice were further processed for histological evaluation. In this regard, periodic acid-Schiff (PAS) staining showed normal and comparable histology of glomeruli and tubulointerstitium in kidneys from KO and control mice (Figure 5A). The expression patterns and levels of podocyte specific markers like podocin and Wilms’ tumor 1 (WT1) were also not different in kidneys from control and KO mice, as assessed by immunoblot analysis of isolated glomeruli (Figure 5B) and immunofluorescence staining (Figure 5C). The number of podocytes per glomerulus, as estimated by absolute counting of WT1 positive podocytes in each glomerulus, was also comparable between control and KO mice (Figure 5D). Transmission electron microscopy of glomeruli demonstrated an ultrastructure of glomeruli and podocytes similar in KO and control mice with normal glomerular filtration barrier and podocyte foot processes. Of note, compared with the control group, glomerular podocytes in KO mice contained significantly more glycogen particles, which were mainly located to the cellular body and major processes of glomerular podocytes, closely associated with the network of tubules of the smooth endoplasmic reticulum, and appeared as electron-dense particles ranging in size from 20 to 25 nm in diameter (Figure 5E). Absolute counting of glycogen particles corroborated the morphologic observations and showed that the amount of measurable glycogen particles was augmented in glomerular podocytes in KO mice (Figure 5F).

### Podocyte specific knockout of GSK3β attenuates proteinuria and ameliorates glomerular injury in murine models of glomerulopathy induced by protein overload

**Figure 6:**
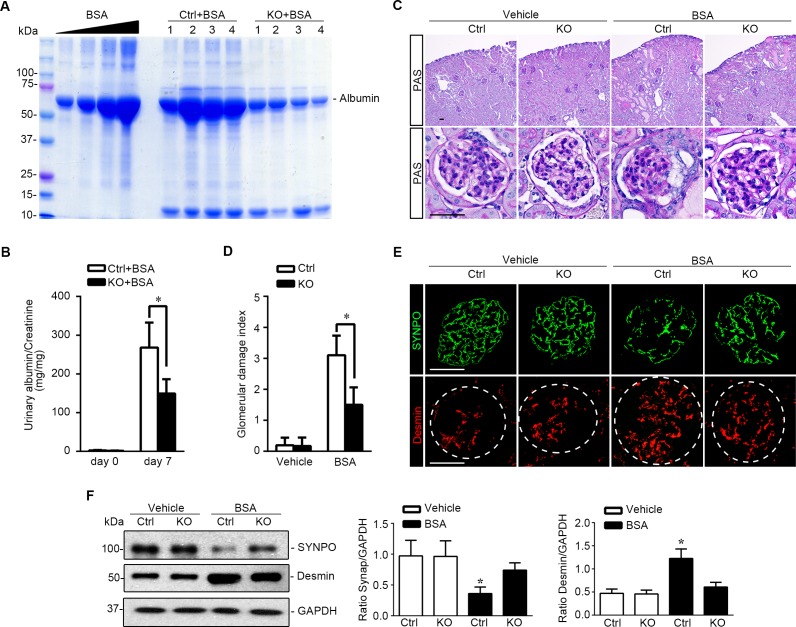
Podocyte specific knockout of GSK3β attenuates proteinuria and ameliorates oxidative glomerular injury in protein overloaded mice **A.** Oxidative glomerular injury was induced in mice by daily i.p. injection of bovine serum albumin as a way of protein overload. Spot urine was collected on day 7 and urine samples (0.5μl) were subjected to SDS-PAGE followed by Coomassie Brilliant Blue staining. BSA 5, 10, 20 and 40μg, served as standard control. **B.** Quantification of urine albumin levels adjusted with urine creatinine concentrations. *P <0.01 (*n* = 6, unpaired *t*-test). **C.** Representative micrographs demonstrate PAS staining and fluorescent immunohistochemistry staining of mouse kidneys procured on day 7 for indicated molecules. PAS staining shows typical renal lesions of proteinuric glomerulopathy, including podocytic swelling and vacuolization, glomerular synechiae, mesangial matrix expansion and protein casts in dilated cortical tubules, which were found 7 days following protein overload. These lesions were significantly attenuated in kidneys from KO mice. Scale bar = 40μm. **D.** Morphometric scoring of glomerular damage index on PAS stained kidney sections prepared on day 7. *P <0.01 (*n* = 6, unpaired *t*-test). **E.** Frozen kidney sections procured on day 7 were subjected to immunofluorescence staining for podocyte specific markers, such as SYNPO, and podocyte injury marker Desmin. Scale bar = 40μm. **F.** Glomeruli were isolated from kidneys from differently treated animals by the magnetic beads-based approach and were homogenized for immunoblot analysis for SYNPO, Desmin, and GAPDH. Relative abundance of SYNPO (middle panel) and Desmin (right panel) expressed in isolated glomeruli from Ctrl and KO mice treated with vehicle (white bar) or BSA (black bar) as determined by densitometric analyses of immunoblot. *P <0.05 *versus* all other groups, (*n* = 3, ANOVA followed by LSD test).

To evaluate the effect of podocyte specific knockout of GSK3β on podocyte pathology, we adopted the murine model of protein overload induced glomerular injury, which recapitulates key features of proteinuric glomerulopathy in human chronic kidney disease, including glomerular hyperperfusion, podocyte injury, massive proteinuria, and glomerulosclerosis [23, 24]. KO mice and control littermates received daily intraperitoneal (i.p.) injection of bovine serum albumin (BSA) consecutively for 7 days with increasing doses (2, 4, 6, 8, 10, 10, 10 mg/g body weight) as a way of protein overload. In control mice, massive albuminuria was evident on day 7, as estimated by urine protein electrophoresis followed by Coomassie Brilliant Blue staining (Figure 6A) and quantified by measuring the urine albumin to creatinine ratios (Figure 6B). This was associated with typical renal lesions of nephrotic glomerulopathy, including podocytic swelling and vacuolization, glomerular synechiae, mesangial matrix expansion and prominent protein casts in dilated cortical tubules, shown by PAS staining (Figure 6C) and quantitated by morphometric scoring of glomerular damage index (Figure 6D). Moreover, signs of podocytopathy were evidently observed in kidney tissues from control mice after protein overload, characterized by the loss of podocyte specific marker synaptopodin and by increased expression of podocyte injury marker Desmin, as shown by fluorescent immunohistochemistry staining (Figure 6E) and by immunoblot analysis of isolated glomeruli (Figure 6F). In stark contrast, KO mice excreted much less albuminuria (Figure 6A and 6B) and presented much less kidney injury, including glomerular damage and protein casts (Figure 6C and 6D). The loss of podocyte marker synaptopodin was attenuated and increased expression of podocyte injury marker Desmin in glomerulus was mitigated in KO kidneys (Figure 6E and 6F), consistent with a lessened podocytopathy.

### Mice with podocyte specific ablation of GSK3β demonstrate a reinforced Nrf2 antioxidant response in glomerulus upon protein overload

**Figure 7:**
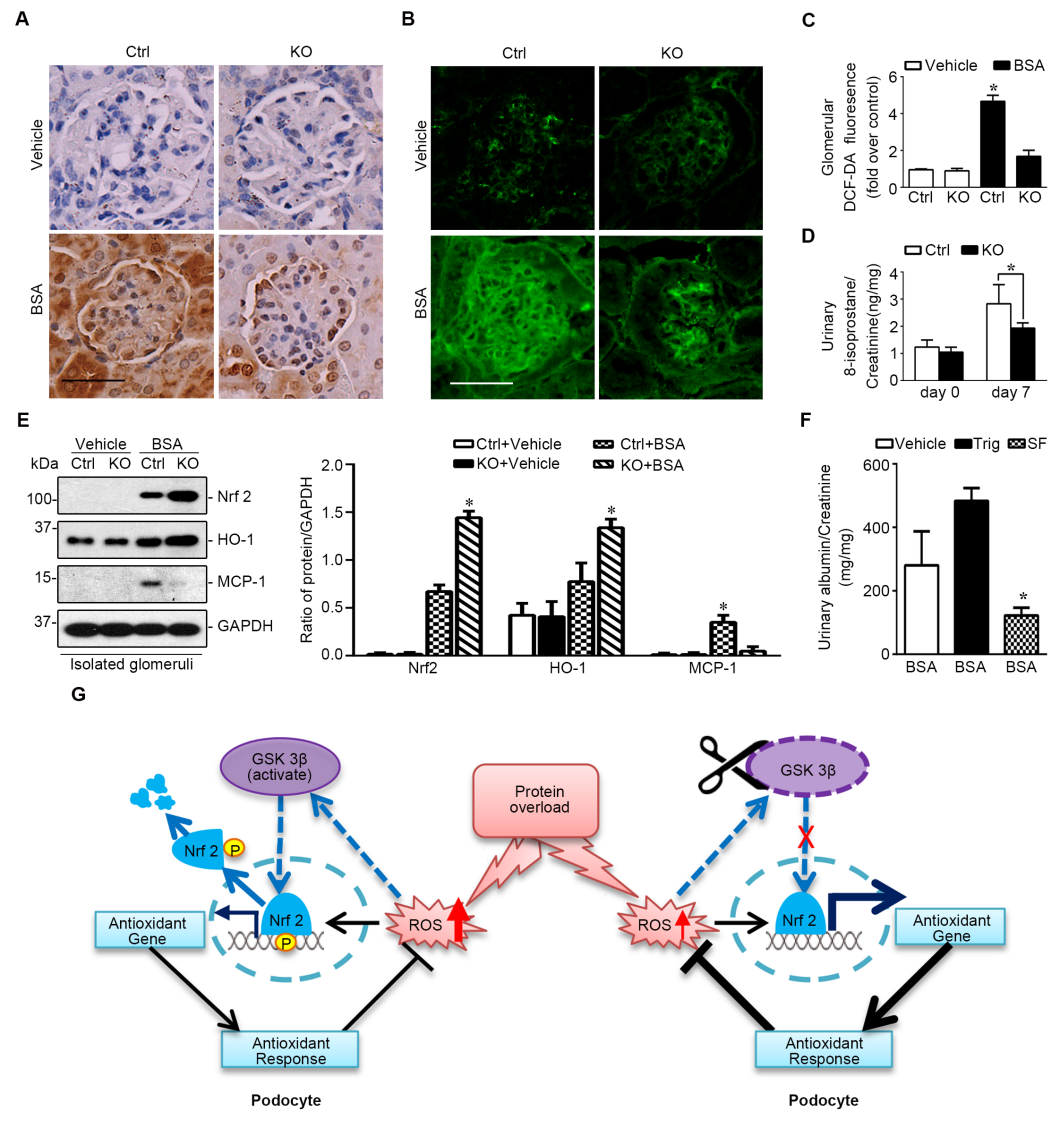
Mice with podocyte specific ablation of GSK3β demonstrate a reinforced Nrf2 antioxidant response in glomerulus upon protein overload Mice were treated as stated in Figure 6. **A.** Representative micrographs of peroxidase immunohistochemistry staining of mouse kidneys procured on day 7 for Nrf2. Scale bar = 40μm. **B.** Fresh renal cortical tissues were obtained from the differently treated animals and prepared as frozen cryostat sections for staining of DCF-DA (green), a marker of ROS. Scale bar = 40μm. **C.** Computerized morphometric analysis of DCF-DA staining in the glomerulus in different groups. Data are expressed as the relative integrated pixel density of DCF-DA fluorescence in the glomerulus as fold induction over the control group. *P < 0.01 *versus* all other groups (*n* = 3, ANOVA followed by LSD test). **D.** Spot urine was collected on day 0 and day 7 from the Ctrl and KO mice treated with BSA. Urine samples were subjected to the analysis of 8-isoprostane levels adjusted with urine creatinine concentrations. *P <0.05 (*n* = 4, unpaired t-test). **E.** Glomeruli were isolated from differently treated animals by the magnetic beads-based approach and were homogenized for immunoblot analysis for Nrf2, HO-1, MCP-1 and GAPDH. Relative abundance of podocin and Nrf2, HO-1 and MCP-1 expressed in isolated glomeruli from differently treated mice. *P <0.01 *versus* all other groups (*n* = 3, ANOVA followed by LSD test). **F.** Male wild type FVB mice were treated with BSA and received i.p. injection of Trig (1mg/kg), SF (12.5 mg/kg) or vehicle every the other day. Spot urine was collected on day 7 and urine samples were subjected to measurement of albumin levels adjusted with urine creatinine concentrations. *P <0.01 *versus* all other groups (*n* = 3, ANOVA followed by LSD test). **G.** Schematic diagram depicts the role of the GSK3β regulated podocyte Nrf2 antioxidant response in oxidative glomerular injury induced by protein overload. (Left panel) In progressive chronic kidney disease, proteinuria induces robust oxidative stress in glomerular podocytes, which switches on the endogenous antioxidant self-defense by activating the Nrf2 dependent response. GSK3β plays a key role in switching off the endogenous antioxidant self-defense through facilitating nuclear export and degradation of Nrf2. As a redox-sensitive signaling transducer, the activity of GSK3β will be amplified upon oxidative stress and GSK3β overactivity facilitates nuclear export and degradation of Nrf2, resulting in switch-off the Nrf2 antioxidant response and aggravated podocyte injury. (Right panel) Genetic targeting of GSK3β in podocytes intercepts nuclear export of Nrf2, reinforces the antioxidant self-defense and ameliorates glomerular injury.

Recent evidence suggests that GSK3β plays a key role in switching off the endogenous antioxidant self defense through facilitating nuclear exclusion and degradation of Nrf2 [11–13]. To explore if an altered Nrf2 antioxidant response exists in glomerular podocytes in KO mice and is responsible for the beneficial effect of podocyte specific GSK3β knockout in protein overload induced glomerulopathy, the expression of Nrf2 was examined. Shown in Figure 7A, immunoperoxidase staining of Nrf2 was very faint in glomerulus in KO and control mice under physiological condition. Following kidney injury induced by protein overload, Nrf2 staining was mildly elevated in glomeruli in control mice, concomitant with a robust oxidative stress in both glomerulus and renal tubules, as indicated by staining with the green fluorescence of the ROS marker, 2′, 7′-dichlorofluorescein-diacetate (DCF-DA) (Figure 7B) combined with morphometric analysis (Figure 7C), and quantitative measurement of the oxidative marker 8-isoprostane in urine (Figure 7D). In KO mice, glomerular staining and nuclear accumulation of Nrf2 was much more enhanced than in control littermates, with a pattern of nuclear distribution in the periphery of glomerular tufts, consistent with podocyte localization. This was accompanied with diminished ROS accumulation in glomeruli (Figure 7B, 7C) and less 8-isoprostane in urine (Figure 7D) in KO mice. The immunohistochemistry staining of Nrf2 was further validated by immunoblot analysis of isolated glomeruli for Nrf2. In parallel with the enhanced expression and nuclear accumulation of Nrf2 in glomeruli in KO mice upon albumin overload, glomerular expression of heme oxygenase-1 (HO-1), a typical Nrf2 target molecule, was promoted, whereas glomerular expression of monocyte chemoattractant protein-1 (MCP-1), a prototype of proinflammatory chemokines, was blunted in KO mice, inferring a reinforced antioxidant and anti-inflammatory response in glomeruli in KO mice (Figure 7E). In addition, concordant with the attenuated proteinuria and less tubular injury and less oxidative stress in KO mice after protein overload, Nrf2 staining in renal tubules was much less intense in KO mice as compared with their control littermates (Figure 7A). The potentiated Nrf2 response seems to be responsible for the attenuated proteinuria and improved glomerular injury in protein overloaded KO mice, because blockade of Nrf2 by trigonelline hydrochloride (Trig) worsened, whereas selective Nrf2 activation by L-sulforaphane (SF) ameliorated proteinuria in this model (Figure 7F).

## DISCUSSION

Glomerular podocytes are highly specialized cells with a complex cytoarchitecture [25]. They are equipped with interdigitated foot processes that envelop the capillaries of the glomeruli in the kidney and are bridged by the slit diaphragm [26]. As a cornerstone of the glomerular filtration barrier, podocytes play a pivotal role in controlling glomerular permselectivity, and preventing protein in the bloodstream from leaking into the urine [25, 27–29]. Injury to podocytes leads to podocytopathy, such as minimal change disease and FSGS in clinical patients, which are commonly manifested as proteinuria, a hallmark of most glomerular diseases. The pathogenic mechanism of podocytopathy remains to be elucidated and has been an intense focus of research. Recent studies from our and other groups suggest that GSK3β might be involved in podocyte injury [15–18, 30]. GSK3β is a redox sensitive multitasking kinase, situated at the nexus of a multitude of signaling pathways, including Wnt, NFκB and more [1, 8–10]. However, the exact role of GSK3β in podocyte injury is still debated among researchers because various studies exploiting selective small molecule inhibitors of GSK3β reached conflicting conclusions. For instance, inhibition of GSK3β by the selective small molecule inhibitor 6-bromoindirubin-3′-oxime (BIO) at a low dose dramatically normalized proteinuria and attenuated histologic injury of glomeruli in rat models of diabetic nephropathy, although hyperglycemia was not corrected, implying direct antiproteinuric and renoprotective action [31]. However, Matsui et al. found that high-dose BIO exacerbated proteinuria and loss of glomerular nephrin in puromycin-injured rats [32]. Another study by Dai et al. reported that a transient and low level of proteinuria followed by a rapid spontaneous remission was provoked by an ultrahigh dose of lithium chloride (16mmol/kg), which is almost two times the median lethal dose of lithium chloride in mice [33]. In stark contrast, our study demonstrated that inhibition of GSK3β by low-dose lithium conferred prominent protection against podocyte injury in mice with adriamycin nephropathy [15, 16, 30]. The most likely explanation for these conflicting findings might be the drawbacks in the nature of the chemical inhibitors. As typical kinase blockades, GSK3β inhibitors, including lithium, 6-bromoindirubin-3′-oxime, SB216763, and 4-benzyl-2-methyl-1, 2, 4-thiadiazolidine-3, 5-dione, raise concerns of selectivity, specificity and off target action, particularly at high doses. In addition, podocytopathy has a multifactorial etiology involving both podocyte direct injury and systemic immune related mediators. Recent evidence indicates that humoral factors, such as circulating permeability factors or lymphotoxins, are an important cause of podocytopathy in human [34]. In animal models of podocyte injury, manipulation of systemic immunity alters the risk and severity of podocyte injury and proteinuria, denoting the contribution of immune dysregulation to podocytopathy [35]. Therefore, putative systemic effects of GSK3β inhibitors might confound their direct and primary actions on podocytes in animal models of podocyte injury. To avoid the above concerns of systemic use of chemical inhibitors, podocyte specific knockout would be an ideal approach to decipher the role of GSK3β in podocyte injury and was successfully achieved in this study in adult mice by employing the doxycycline inducible Cre/*lox*P gene targeting system.

Inducible deletion of GSK3β in mature glomerular podocytes seems to generate no gross phenotype and result in normal kidney physiology and histology, denoting that GSK3β is dispensable for podocyte function and structural integrity under physiologic condition. This is consistent with clinical observation that inhibition of GSK3β by lithium in human is largely safe and rarely causes glomerular injuries. As the mainstay of therapy for bipolar affective disorder in the past fifty years, lithium, a typical inhibitor of GSK3β, usually needs to be used at the psychiatric high dose for a long time (usually >10 years). Although a few of case series of lithium related renal adverse effects have been reported, including proteinuria, nephrotic syndrome and glomerular disease, it remains controversial if the glomerulopathy is caused by lithium *per se* or by a coincident glomerular disease [36]. Moreover, according to a large-scale epidemiology study, the incidence of chronic kidney disease and proteinuria in psychiatric patients receiving long term lithium therapy is actually comparable with that in the general population [37], suggesting that the GSK3β inhibitor, lithium, is unlikely toxic to glomerular cells, like podocytes.

Under diseased state, GSK3β has been implicated in multiple pathogenic or self-defense pathways, such as the self-protective Nrf2 antioxidant pathway. Nrf2, is a transcription factor that upon oxidative stress transactivates a broad spectrum of enzymes involved in antioxidation, detoxification, cell survival, anti-inflammatory response and more. Nrf2 serves as the master regulator of detoxification/antioxidant response as one of the most crucial endogenous measures for self-protection. Under normal conditions, Nrf2 is sequestrated in the cytoplasm by Keap1 and thereby is constantly degraded. In response to stress, Nrf2 is liberated and translocated into nucleus to initiate its transcriptional activity (Figure 7G). Recent data from our and other groups indicate that Nrf2 antioxidant pathway is also regulated by the redox sensitive GSK3β [11–13, 38]. Nrf2 encompasses multiple GSK3β phosphorylation consensus motifs, serves as a cognate substrate for GSK3β and are subjected to GSK3β-directed phosphorylation [14]. Phosphorylation of Nrf2 by GSK3β facilitates Nrf2 nuclear exit and proteasomal degradation, thus represents a delayed mechanism that controls switching off Nrf2 activation (Figure 7G). The glomerular Nrf2 activity in normal kidneys is very low, as shown by immunohistochemistry staining in this and other [39] study, but following oxidative stress induced by albumin overload is augmented in the periphery of glomerular tufts, indicative of a pattern of podocyte distribution. This injury induced Nrf2 expression was significantly reinforced in GSK3β knockout mice. Our findings suggest that targeted inhibition of GSK3β does not trigger Nrf2 activation under normal condition, but potentiate the inducibility of Nrf2 response upon oxidative stress. This action is totally different from that of the Nrf2 activators like bardoxolone, which recently failed the clinical trial for treating chronic kidney disease in diabetic patients, due to severe adverse effects [40].

In summary, we have successfully developed a line of mice with somatic ablation of GSK3β in podocytes. The mice have no phenotype under physiological conditions, except augmented glycogen accumulation in podocytes. Upon oxidative glomerular injury elicited by protein overload, KO mice demonstrated less proteinuria and attenuated glomerular and podocyte injury, associated with a potentiated induction of the Nrf2 antioxidant response in glomerular podocytes. Our data suggest that GSK3β in podocytes is likely dispensable for the biophysiology of normal glomeruli, but might repress the endogenous antioxidant response upon oxidative glomerular injury.

## MATERIALS AND METHODS

### Animal studies

Animal studies were approved by the institution's Animal Care and Use Committee and they conformed to the United States Department of Agriculture regulations and the National Institutes of Health guidelines for humane care and use of laboratory animals.

### Generation of the doxycycline-inducible podocyte-specific GSK3β knockout mice and genotyping

Mice with the floxed *GSK3β* gene (*GSK3β^fl/+^*) were kindly provided by Dr James Woodgett (Samuel Lunenfeld Research Institute of Mount Sinai Hospital, Toronto, Canada) and had been generated by introducing *lox*P sites upstream and downstream of exons 2 on a genetic background of C57/B6J [41]. *GSK3β^fl/+^* mice were backcrossed for ten generations into an FVB/N congenic background. *TRE^cre^* mice on a genetic background of FVB/N (Jackson Laboratory, Bar Harbor, ME) contain a Cre recombinase cassette under control of the *tet*-operator. *NPHS2^rtTA^* mice on a genetic background of FVB/N (Jackson Laboratory) produce the rtTA protein under control of a *NPHS2* promoter fragment. To generate doxycycline-inducible podocyte-specific GSK3β knockout mice (*NPHS2^rtTA^/TRE^cre^/GSK3β^−/−^*, KO), *GSK3β^fl/+^* mice were crossed with *TRE^cre^* mice and *NPHS2^rtTA^* mice, respectively. Then, *TRE^cre^/GSK3β^fl/+^* mice cross with *NPHS2^rtTA^/GSK3β^fl/+^* mice. All littermates lacking the *Cre* transgene served as a control. At 8 to 10 weeks old, mice received doxycycline (TCI, Tokyo, Japan) treatment *via* drinking water (2mg/ml with 5% sucrose, protected from light) for a total of 14 days to induce podocyte-specific GSK3β deletion. A routine PCR protocol was used for genotyping tail DNA samples with the following primer pairs: *rtTA* transgene genotyping, forward: 5′-GAA-CAA-CGC-CAA-GTC-ATT-CCG-3′ and reverse: 5′-TAC-GCA-GCC-CAG-TGT-AAA-GTG-G-3′, which generated a 196-bp fragment; and *Cre* transgene genotyping, forward: 5′-AGG-TGT-AGA-GAA-GGC-ACT-TAG-C-3′ and reverse: 5′-CTA-ATC-GCC-ATC-TTC-CAG-CAG-G-3′, which generated a 411-bp fragment; and *GSK3β* genotyping, forward: 5′-GGG-GCA-ACC-TTA-ATT-TCA-TT-3′ and reverse: 5′-GTG-TCT-GTA-TAA-CTG-ACT-TCC- TGT-GGC-3′, which yielded 685- and 585-bp bands, respectively, for the floxed and wild-type alleles. All animals were born normally at the expected Mendelian frequency.

### Animal model of protein overload

The mouse model of protein overload was established by intraperitoneal (i.p.) injection of BSA as previously described [42, 43]. KO mice and control littermates received endotoxin-free BSA (Sigma, St. Louis, MO) (250mg/ml, dissolved in PBS) or an equal volume of PBS intraperitoneally for 7 consecutive days with increasing doses (2, 4, 6, 8, 10, 10, 10 mg/g body weight). Additional wild type FVB mice were treated with endotoxin-free BSA *via* i.p. injection as described above and received i.p. injection of Trig (1mg/kg, Sigma), SF (12.5 mg/kg, Sigma) or vehicle every the other day. Spot urine was collected before injection and 7 days after first injection and subjected to urinary albumin assay adjusted with urine creatinine concentrations. Proteinuria was confirmed by urine electrophoresis followed by Coomassie Brilliant Blue staining. Mice were sacrificed on day 7.

### Histology and immunohistochemical staining

Formalin-fixed mouse kidney were embedded in paraffin and prepared in 3μm-thick sections. Sections were processed for PAS staining. The morphologic features of all the sections were assessed by a single observer in a blinded manner. A semiquantitative glomerular damage index was used to evaluate the degree of glomerular damage [44]. The severity of injury for each glomerulus was graded from 0 to 5 as follows: 0 represents no lesions; 1, damage of <20% of the glomerulus; and 2, 3, 4, and 5, damage of 20% to 40%, >40% to 60%, and >60% to 80%, and >80% of the glomerulus, respectively. A whole-kidney average glomerular damage index was obtained by averaging scores from all glomeruli on one section [45–47]. Immunoperoxidase staining was performed with a Vectastain ABC kit (Vector Laboratories, Burlingame, California, USA) by using of primary antibodies against GSK3α (Santa Cruz Biotechnology, Santa Cruz, CA), GSK3β (Cell Signaling, Danvers, MA), and Nrf2 (Santa Cruz). As a negative control, the primary antibody was replaced by preimmune IgG from the same species and no specific staining was noted.

### Glomerular isolation and primary culture of podocytes

Isolation of glomeruli from the GSK3β KO mice and control mice was performed as described previously [48, 49]. In brief, mice were anesthetized and the kidney was perfused with 5ml of phosphate-buffered saline containing 8×10^7^ Dynabeads M-450 (Dynal Biotech ASA, Oslo, Norway). The kidneys were then cut into 1 mm^3^ pieces and digested in collagenase A. The tissue was then press gently through a 100μm cell strainer (Falcon, Bedford, MA,) and glomeruli-containing Dynabeads were gathered using a magnetic particle concentrator. The enriched glomeruli were plated on collagen type I-coated dishes at 37°C in RPMI 1640 medium (Life Technologies, Grand Island, NY) with 10% fetal bovine serum(Life Technologies), 0.075% sodium bicarbonate (Sigma), 1mM sodium pyruvate (Sigma), 100U/ml penicillin and 100μg/ml streptomycin (Life Technologies) in a humidified incubator with 5% CO_2_. Subculture of primary podocytes was performed by detaching the glomerular cells with 0.25% trypsin-EDTA (Invitrogen, Carlsbad, CA), followed by sieving through a 40-μm cell strainer (Falcon), and culture on collagen type I-coated dishes. Podocytes of passages 1 or 2 were characterized by the expression of multiple podocyte specific markers and used in all experiments.

### Western immunnoblot analysis

Cultured cells were lyzed and animal tissues homogenized in radioimmunoprecipitation assay supplemented with protease inhibitors and samples were processed for immunoblot analysis. The antibodies against GSK3α/β, podocin, WT1, synaptopodin (SYNPO), Nrf2, Desmin, HO-1, MCP-1, glyceraldehydes-3-phosphate dehydrogenase (GAPDH), were purchased from Santa Cruz Biotechnology.

### Immunofluorescence staining

Cryosection of kidneys were fixed with 4% paraformaldehyde (Sigma), permeabilized and stained with primary antibodies against GSK3β (Cell Signaling), SYNPO (Santa Cruz), Podocin (Santa Cruz), WT1 (Santa Cruz), Desmin (Santa Cruz), followed by Alexa fluorophore-conjugated secondary antibody staining (Life Technologies). Finally, sections were counterstained with 4′,6-diamidino-2-phenylindole (DAPI), mounted with Vectashield mounting medium (Vector Laboratories), and visualized using a fluorescence microscope (BX43, Olympus, Tokyo, Japan) or a Zeiss LSM710 Meta confocal microscope (Carl Zeiss AG, Cologne, Germany). For dual-color staining, images were acquired sequentially to avoid dye interference. ImageJ software was used for post-processing of the images, e.g., scaling and merging.

### Urinary and serum analyses

To discern the protein compositions in urine, equal amounts of urine samples were subjected to SDS-PAGE followed by Coomassie Blue (Sigma) staining. Urine albumin concentration was measured using a mouse albumin enzyme-linked immunosorbent assay quantitation kit (Bethyl Laboratories Inc., Montgomery, TX). Urine and serum creatinine concentration was measured by a creatinine assay kit (BioAssay Systems, Hayward, CA). Urinary 8-isoprostane levels were determined using a commercial kit as per the manufacture's protocol (Cayman Chemical Company, Ann Arbor, MI).

### Transmission electron microscopy

For transmission electron microscopy, kidney cortical tissues were cut into small pieces (1 mm^3^), fixed with 2.5% glutaraldehyde, and embedded in Epon 812 (Polysciences Inc., Warrington, PA). Conventional electron micrographs were obtained using an EM-10 microscope (Zeiss) operated at 60 kV. Absolute counting of total measurable glycogen particles was performed in 10 random electron microscopy fields of glomerular podocytes per mouse in 6 mice per group.

### Detection of reactive oxygen species (ROS) generation by fluorescence

The production of ROS in the mouse kidney was evaluated by detecting the fluorescence intensity of DCF-DA (Sigma) as described previously [50–53]. In brief, fresh kidney cryostat sections were incubated with 10μm DCF-DA in a light-protected humidified chamber at 37°C for 30 min, subsequently washed twice with PBS for 5 min, mounted with mounting medium and visualized with a fluorescence microscope. At least three fields were randomly chosen from each kidney sections and 5~10 glomeruli in each field were analyzed for morphometric quantification of the fluorescence signal by using ImageJ software. Results were expressed as fold change relative to control.

### Statistical analysis

For immunoblot analysis, bands were scanned and the integrated pixel density was determined using a densitometer and the ImageJ analysis program. All data are expressed as means ± SD or as otherwise indicated. All immunoblot analyses were independently repeated three to four times. Statistical analysis of the data from multiple groups was performed by one-way analysis of variance (ANOVA) followed by Fisher's Least Significant Difference (LSD) tests. Data from two groups were compared by Student's t-test. *P* < 0.05 was considered significant.
